# Impact of X‐ray beam quality on organ‐absorbed dose in chest CT: Evaluation using spectral analysis and beam‐shaping filters

**DOI:** 10.1002/acm2.70754

**Published:** 2026-08-18

**Authors:** Yusuke Ito, Yusei Nishihara, Masanao Kobayashi, Tomoya Hibino, Yasuki Asada

**Affiliations:** ^1^ Department of Radiological Technology Daiyukai Daiichi Hospital Ichinomiya Aichi Japan; ^2^ Graduate School of Medical Sciences Fujita Health University Toyoake Aichi Japan; ^3^ Department of Radiology Fujita Health University Hospital Toyoake Aichi Japan; ^4^ Department of Medical Imaging Technology Clinical Collaboration Unit Faculty of Medical Sciences, Fujita Health University Toyoake Aichi Japan; ^5^ Department of Medical Technology General Daiyukai Hospital Ichinomiya Aichi Japan; ^6^ Faculty of Radiological Technology School of Medical Sciences Fujita Health University Toyoake Aichi Japan

**Keywords:** beam quality, beam‐shaping filter, chest CT, dosimetry, spectral analysis, X‐ray spectrum

## Abstract

**Background:**

In computed tomography (CT), radiation dose is commonly evaluated using system‐displayed metrics such as CTDIvol; however, variations in X‐ray beam quality may influence organ absorbed dose even under identical CTDIvol conditions.

**Purpose:**

To investigate the impact of X‐ray beam quality, including tube voltage and beam‐shaping filters, on organ absorbed dose in chest computed tomography (CT) under identical CTDIvol conditions, with particular emphasis on spectral characteristics and energy‐dependent dose conversion.

**Methods:**

Half‐value layer (HVL) measurements were performed using the aluminum attenuation method to determine effective energy under different tube voltage conditions (80, 100, 120, and 135 kV, and 120 kV with a silver [Ag] filter). X‐ray spectra were measured using a CdTe‐based spectrometer with a 90°Compton scattering configuration, and mean photon energy was calculated from the measured spectra. Absorbed doses in the breast and lung were evaluated using optically stimulated luminescence (OSL) dosimeters placed in an anthropomorphic phantom. Scan parameters were adjusted to maintain a constant CTDIvol (6.2 mGy) across all conditions. Absorbed dose was calculated from air kerma using f‐factors derived from both effective energy and mean photon energy.

**Results:**

Effective energy increased with tube voltage and was markedly elevated by the Ag filter. Spectral measurements demonstrated substantial reduction of low‐energy photons and beam hardening under the Ag condition. Despite identical CTDIvol, both air kerma and absorbed dose varied with beam quality. Absorbed dose generally increased with effective energy in both tissues; however, under the 120 kV + Ag condition, the two tissues diverged: the lung absorbed dose decreased despite the higher effective energy, whereas the breast absorbed dose continued to increase. When the mean photon energy was used instead, this discrepancy was resolved differently: the absorbed dose decreased under the 120 kV + Ag condition in both the breast and the lung. This tissue‐dependent sensitivity to the choice of energy metric was attributable to the energy dependence of the f‐factor, which approached or exceeded unity in the breast under the Ag condition.

**Conclusions:**

Organ absorbed dose in chest CT varies with beam quality even under identical CTDIvol conditions, and this variation is tissue‐dependent. Effective energy and mean photon energy can lead to different, and in the breast even opposite, conclusions regarding the direction of dose change under the same irradiation condition. Specifically, under standard (non‐filtered) conditions, mean‐photon‐energy‐based absorbed doses in the breast were approximately 4%‐5% higher than effective‐energy‐based estimates, whereas under the 120 kV + Ag condition this relationship reversed, with mean‐photon‐energy‐based doses approximately 1.6% lower. Although the magnitude of this reversal was relatively small, it was consistent and reproducible across repeated measurements. These findings highlight that the choice of energy metric used to convert air kerma to absorbed dose can meaningfully affect organ dose assessment in CT dosimetry, particularly when the f‐factor approaches or exceeds unity.

## 1 INTRODUCTION

X‐ray computed tomography (CT) is an indispensable imaging modality for clinical diagnosis; however, optimization of patient radiation dose remains a critical issue. In current dose management, dose metrics displayed by CT systems, such as the volume CT dose index (CTDIvol) and dose‐length product (DLP), are widely used as indicators, and dose optimization is commonly performed based on diagnostic reference levels (DRLs).^1^, ^2^, ^3^ Traditionally, dose reduction in CT examinations has primarily been achieved by adjusting the tube current (mA) or tube current‐time product (mAs), whereas the tube voltage has generally been fixed.

In recent years, dose optimization strategies incorporating adjustments in both tube current and tube voltage have become increasingly prevalent.^4^, ^5^, ^6^ The introduction of low‐tube‐voltage imaging and automatic tube‐voltage selection systems has enabled the simultaneous achievement of dose reduction and maintenance of image quality. Furthermore, modern CT systems allow the modification of X‐ray beam quality through the use of beam‐shaping filters, such as silver (Ag) filters, which selectively remove low‐energy components of the X‐ray spectrum.^7^

However, even under identical CTDIvol conditions, differences in tube voltage and beam‐shaping filters can alter the effective energy and spectral distribution of X‐rays. Consequently, the attenuation and scattering behavior of X‐rays within the patient may change, and the distribution of absorbed dose among different tissues and organs may not be equivalent.^8^, ^9^ In particular, differences in absorbed dose distribution related to beam quality variations have been reported, especially between superficial and deeper regions within the body.^10^ In addition, the f‐factor used to convert air kerma to absorbed dose is dependent on photon energy;^8^, ^9^ therefore, evaluations based solely on effective energy may not adequately reflect the actual X‐ray spectrum.

Although previous studies have reported that differences in tube voltage and beam quality affect the organ‐absorbed dose, it remains unclear how variations in effective energy and mean photon energy arising from differences in tube voltage and the use of beam‐shaping filters, including silver filters, affect absorbed doses in the breast and lungs during chest CT under identical CTDIvol conditions.^10^

Therefore, the purpose of this study was to investigate the impact of differences in effective energy and mean photon energy resulting from variations in tube voltage and the presence or absence of a silver filter on the absorbed doses in the breast and lungs during chest CT. The evaluation was performed using an anthropomorphic phantom and direct dose measurements. Although the energy dependence of the f‐factor and the influence of beam‐shaping filters on organ dose have each been discussed separately in previous reports, this study combines direct organ‐dose measurement with simultaneous spectral measurement under identical CTDIvol conditions, which allows the effective energy and the mean photon energy to be compared directly rather than estimated independently. This approach reveals that the two energy metrics can lead to opposite conclusions regarding the direction of dose change in a specific tissue (the breast) under the same irradiation condition, a tissue‐dependent discrepancy that, to our knowledge, has not been demonstrated experimentally in previous work.

## 2 METHODS

### 2.1 CT system

A CT scanner (Aquilion ONE PRISM Edition; Canon Medical Systems, Tochigi, Japan) was used. Chest CT was selected as the target examination.

### 2.2 Measurement of half‐value layer and effective energy

To evaluate the X‐ray beam quality under each imaging condition, the half‐value layer (HVL) was measured using the aluminum attenuation method.^11^, ^12^ The measurements were performed using an ionization chamber (Model 9015, Radcal, USA) with a CT ionization chamber (10 × 6‐0.6CT, Radcal, USA), while the X‐ray tube was fixed. The attenuation curves were obtained by placing aluminum plates of varying thicknesses along the beam path.

The effective energy was calculated from the measured HVL.^8^, ^13^, ^14^ It was defined as the monoenergetic photon energy corresponding to the measured attenuation based on the mass attenuation coefficient of aluminum.

### 2.3 Measurement of X‐ray spectrum and mean photon energy

The effective energy represents a monoenergetic approximation; however, actual X‐rays exhibit a continuous spectrum. Therefore, the X‐ray spectra were measured for each tube voltage condition.

X‐ray spectra were measured using an X‐ray spectrum analyzer with a CdTe detector (RAMTEC Type 413; Toyo Medical Co., Japan) and a multichannel analyzer (MCA8000A; Amptek Inc., USA). Compton‐scattered photons at 90° were measured using a carbon rod placed at the isocenter as a scatterer.

**FIGURE 1:**
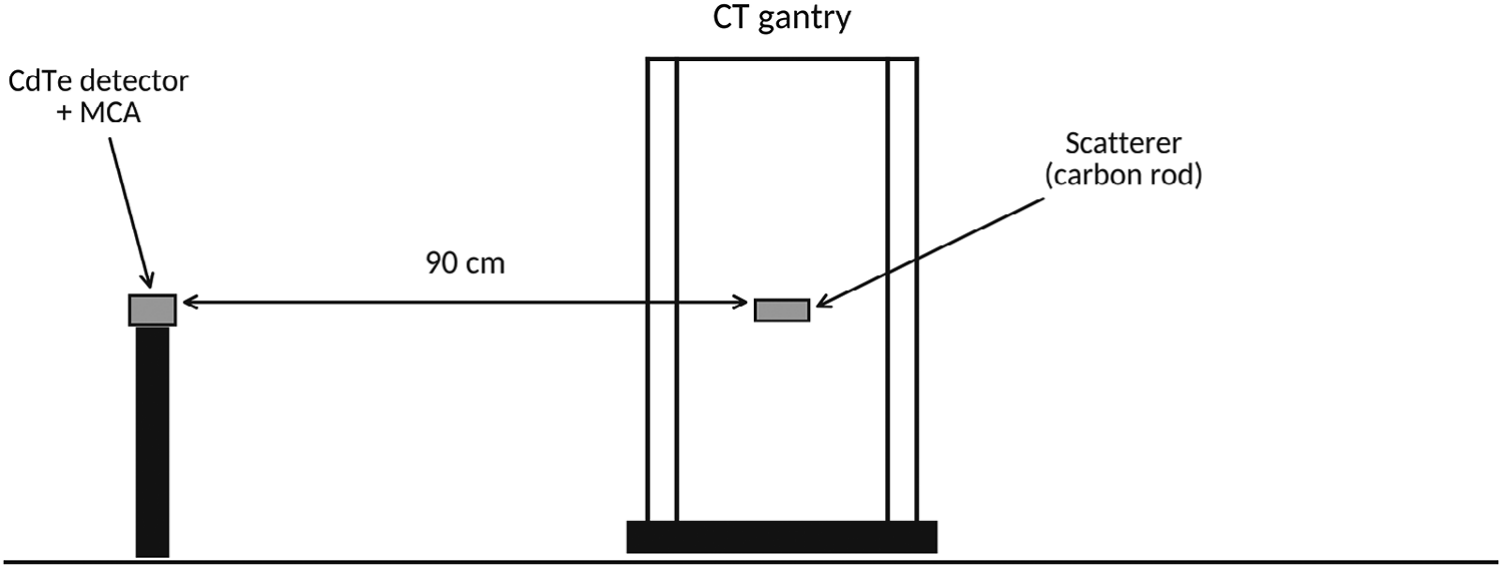
Schematic of the experimental setup for X‐ray spectrum measurement, showing the relative positions of the CT gantry, the carbon rod scatterer at the isocenter, and the CdTe detector with the multichannel analyzer (MCA). Tungsten collimators (not shown) were incorporated into the scatterer and detector assemblies.

The spectrum analyzer was positioned behind the CT gantry at the same height as the isocenter and arranged at 90° relative to the X‐ray beam axis. The distance between the carbon rod and CdTe detector was set to 90 cm. Tungsten collimators (1.6 mm) were placed on both the scatterer and detector sides to optimize beam directionality and minimize pulse pile‐up effects (Figure 1).

The tube voltages were set to 80, 100, 120, and 135 kV. For 120 kV, an additional condition using a silver (Ag) beam‐shaping filter was included, whereas the other conditions used a conventional filter without Ag. The tube current was adjusted such that approximately 2000 photons were detected per scan to avoid detector saturation.

The mean photon energy (E) of the CT spectrum was calculated.^7^ All measurements and energy evaluations were performed in accordance with international standards for radiation characterization.^15^

### 2.4 Phantom and dosimeter

An anthropomorphic phantom (ATOM Model 701; CIRS, Norfolk, VA, USA) was used for the absorbed dose measurements. According to the manufacturer's specifications, the chest cross‐section of this phantom measures approximately 23 cm (anteroposterior) × 32 cm (lateral), representing an average adult body habitus. For breast dose evaluation, a dedicated breast phantom (701‐BR01) was attached.

Optically stimulated luminescence (OSL) dosimeters (NanoDot; Nagase Landauer, Tokyo, Japan) were used for dose measurements, and readings were obtained using a microSTAR OSLD reader (Nagase Landauer, Tokyo, Japan).

Because OSL dosimeters exhibit energy dependence, the calibration factors were determined by simultaneous irradiation of the OSL dosimeters and the ionization chamber at the effective energies calculated in Section 2.2.

The OSL dosimeters were inserted into the phantom at 11 locations within the lungs (single axial slice) and 10 locations within the breasts. Each dosimeter was read five times, and the average of the three values, excluding the maximum and minimum readings, was used as the final value. The air kerma was calculated by multiplying the final reading by the calibration factor of each dosimeter.

### 2.5 Scan protocol

Helical scanning was performed with a fixed beam collimation of 40 mm (0.5 mm × 80 rows), a pitch of 0.813, and a scan range of 300 mm. A calibration field of view of “L” was selected, which determines the bow‐tie filter shape on this CT system. The scanning conditions were adjusted such that the CTDIvol was 6.2 mGy at each tube voltage by modifying the tube current and rotation time as follows: 430 mA and 1.0 s/rotation at 80 kV; 360 mA and 0.5 s/rotation at 100 kV; 200 mA and 0.5 s/rotation at 120 kV; 140 mA and 0.5 s/rotation at 135 kV; and 370 mA and 1.0 s/rotation at 120 kV with the Ag filter. Because the Ag filter substantially attenuates low‐energy photons, a higher tube current and longer rotation time were required to achieve the same CTDIvol as the other conditions. Five imaging conditions were evaluated in total: 80, 100, 120, 135 kV, and 120 kV with a silver beam‐shaping filter. To ensure reproducibility, each scan was performed five times using orbit‐synchronized scanning.

### 2.6 Dose calculation

Air kerma was calculated from OSL dosimeter readings using the following equation:

Air kerma = OSL reading × calibration factor

Absorbed dose for each tissue was calculated as:

Absorbed dose = Air kerma × f‐factor

The f‐factor is defined as the ratio of the mass energy absorption coefficients of the tissue to those of air. Because these coefficients depend on the photon energy, the f‐factors were calculated using both the effective and mean photon energies.^8^, ^9^

This approach enables a comparison between the absorbed doses based on the monoenergetic approximation and those reflecting spectral information. For each condition, measurements were performed five times, and the mean and standard deviation were calculated.

## 3 RESULTS

### 3.1 Half‐value layer and effective energy

Half‐value layer (HVL) and effective energy under each tube voltage condition are summarized in Table 1.

**TABLE 1:** Half‐value layer (HVL) and effective energy (Eeff) under each tube voltage condition.

Tube voltage (kV)	HVL (mm Al)	Effective energy (keV)
80	6.0	46.9
100	7.4	51.9
120	8.5	56.1
135	9.2	58.7
120 + Ag filter	13.7	89.2

The HVL increased with increasing tube voltage, from 6.0 mmAl at 80 kV to 9.2 mmAl at 135 kV. Correspondingly, the effective energy increased from 46.9 keV to 58.7 keV.

In contrast, under the 120 kV condition with a silver (Ag) filter, the HVL markedly increased to 13.7 mmAl, and the effective energy increased to 89.2 keV.

### 3.2 X‐ray spectra

**FIGURE 2:**
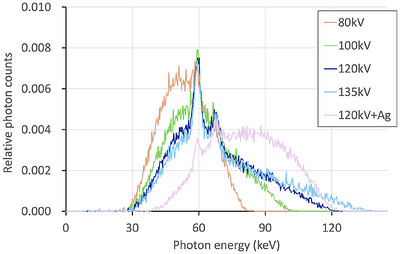
X‐ray spectra under different tube voltage conditions, showing that the spectra shift toward higher energies with increasing tube voltage and that low‐energy components are markedly reduced under the 120 kV + Ag condition due to beam hardening.

X‐ray spectra for each condition are shown in Figure 2.

With increasing tube voltage, the spectra shift toward higher energies. Under the 120 kV + Ag condition, the low‐energy components were substantially reduced, whereas the high‐energy components increased, indicating pronounced beam hardening.

The mean photon energies obtained by integrating each spectrum were 53.96 keV at 80 kV, 60.92 keV at 100 kV, 66.70 keV at 120 kV, 70.90 keV at 135 kV, and 80.97 keV at 120 kV with the Ag filter.

### 3.3 Air kerma and absorbed dose

The air kerma and absorbed doses in the breasts and lungs are presented in Table 2.

**TABLE 2:** Air kerma and absorbed doses (calculated using the effective energy and the mean photon energy) in the breast and lung under each tube voltage condition.

		80 kV	100 kV	120 kV	135 kV	120 kV+Ag
Breast air kerma	Mean (mGy)	8.02	8.44	8.81	8.76	7.98
	SD (mGy)	0.11	0.46	0.39	0.51	0.20
	CV (%)	1.3	5.4	4.5	5.8	2.5
Lung air kerma	Mean (mGy)	9.16	9.99	10.56	10.84	9.97
	SD (mGy)	0.26	0.39	0.43	0.38	0.10
	CV (%)	2.8	3.9	4.1	3.5	1.0
Breast absorbed dose with Eeff	Mean (mGy)	7.02	7.62	8.13	8.22	8.33
	SD (mGy)	0.09	0.41	0.36	0.48	0.21
	CV (%)	1.3	5.4	4.5	5.8	2.5
Lung absorbed dose with Eeff	Mean (mGy)	9.67	10.59	11.24	11.58	10.87
	SD (mGy)	0.20	0.50	0.55	0.49	0.11
	CV (%)	2.0	4.8	4.9	4.2	1.0
Breast absorbed dose with E	Mean (mGy)	7.32	8.03	8.55	8.63	8.20
	SD (mGy)	0.10	0.44	0.38	0.50	0.21
	CV (%)	1.3	5.4	4.5	5.8	2.5
Lung absorbed dose with E	Mean (mGy)	9.74	10.69	11.38	11.72	10.85
	SD (mGy)	0.20	0.49	0.51	0.44	0.12
	CV (%)	2.1	4.5	4.5	3.8	1.1

The air kerma generally increased with increasing tube voltage in both tissues, although the breast air kerma showed a slight decrease at 135 kV (8.76 mGy) compared with 120 kV (8.81 mGy). When the absorbed dose was calculated using the mean photon energy, the values were higher than those calculated using the effective energy at 80, 100, 120, and 135 kV in both tissues. Under the 120 kV + Ag condition, however, the absorbed dose exhibited tissue‐dependent behavior: in the lung, the absorbed dose decreased regardless of which energy metric was used, whereas in the breast, the absorbed dose calculated using the effective energy increased further, while that calculated using the mean photon energy decreased.

Under all the conditions, the values in the lungs were higher than those in the breasts.

To illustrate the practical impact of these findings, the absorbed dose calculated using the effective energy differed from the air kerma measured at the same location by approximately −12.5% to +4.4% in the breast and +5.6% to +9.0% in the lung across the tested conditions, with the largest deviations occurring at 80 kV in the breast and at 120 kV + Ag in the lung. This indicates that relying on the air kerma alone, without accounting for beam quality, could result in an organ dose estimate that differs from the directly measured absorbed dose by approximately 10% or more, depending on the tissue and imaging condition. In comparison, the difference between the absorbed doses calculated using the effective energy and the mean photon energy was smaller in most conditions, ranging from approximately 0.2% to 1.2% in the lung and from −1.6% to +5.4% in the breast.

### 3.4 Relationship between beam quality and dose

**FIGURE 3:**
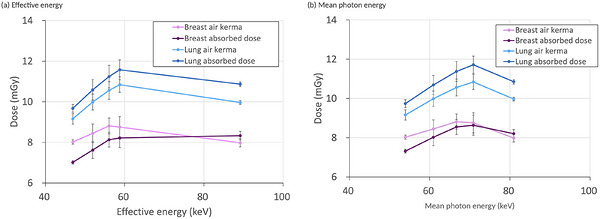
Relationship between (a) effective energy and (b) mean photon energy and the air kerma and absorbed doses, demonstrating that air kerma and absorbed dose generally increase with photon energy, with a tissue‐dependent exception under the 120 kV + Ag condition: the lung absorbed dose decreases despite the higher energy in both panels, whereas the breast absorbed dose increases further when using the effective energy (a) but decreases when using the mean photon energy (b).

The relationships among the effective energy, air kerma, and absorbed dose are shown in Figure 3(a), and the corresponding relationships using the mean photon energy are shown in Figure 3(b).

Both air kerma and absorbed dose tended to increase with increasing effective energy in both tissues (Figure 3(a). However, under the 120 kV + Ag condition, the two tissues showed opposite trends despite the high effective energy: the lung absorbed dose decreased, whereas the breast absorbed dose increased further. When the mean photon energy was used instead (Figure 3(b), the dose generally increased with increasing mean photon energy; however, a decrease was observed under the 120 kV + Ag condition in both the lung and breast. In the lung, similar behavior was observed between the effective‐energy‐ and mean‐photon‐energy‐based calculations, whereas in the breast, the two metrics led to opposite conclusions under this condition, as described above.

### 3.5 Comparison of absorbed dose based on effective energy and mean photon energy

**FIGURE 4:**
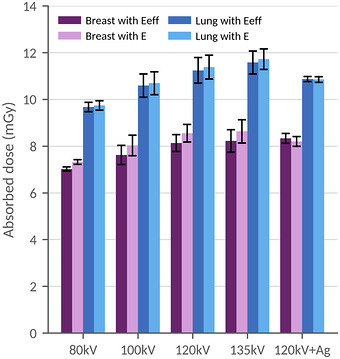
Comparison of absorbed doses (with standard deviation error bars) calculated using effective energy and mean photon energy, indicating differences in dose estimation depending on the energy metric used.

A comparison of the absorbed doses calculated using the effective and mean photon energies is shown in Figure 4. For the breast tissue, the absorbed dose increased when the mean photon energy was used instead of the effective energy at 80, 100, 120, and 135 kV; however, under the 120 kV + Ag condition, the absorbed dose decreased. In contrast, for the lung tissue, the difference between the two energy metrics was smaller than that in the breast. The effective energy, mean photon energy, and the corresponding f‐factors for the breast and lung under each condition are summarized together in Table 3.

**TABLE 3:** Comparison of effective energy, mean photon energy, and the corresponding f‐factors for the breast and lung under each tube voltage condition.

	80 kV	100 kV	120 kV	135 kV	120 kV+Ag
Effective energy (keV)	46.9	51.9	56.1	58.7	89.2
Mean photon energy (keV)	53.96	60.92	66.70	70.90	80.97
Breast f‐factor (Eeff)	0.87	0.90	0.92	0.94	1.04
Breast f‐factor (E)	0.91	0.95	0.97	0.99	1.03
Lung f‐factor (Eeff)	1.07	1.07	1.08	1.08	1.09
Lung f‐factor (E)	1.07	1.08	1.08	1.08	1.09

## 4 DISCUSSION

In this study, OSL dosimeters were used for dose measurements. OSL dosimeters exhibit angular dependence in response to incident radiation. However, in CT examinations, the X‐ray tube rotates around the subject, resulting in irradiation from multiple directions, which is expected to average the effects of angular dependence.

Ichikawa et al. evaluated the influence of dosimeter orientation under CT conditions using an anthropomorphic phantom and reported that variations ranged from −7.3% to +5.4%, which were considered negligible in clinical practice when system uncertainties were considered.^16^ Therefore, the use of OSL dosimeters in this study is considered appropriate for organ dose evaluation, with minimal impact from angular dependence.

### 4.1 Relationship between beam quality (effective energy) and absorbed dose

In the present study, the absorbed dose in the breast increased monotonically with increasing effective energy across all conditions, including the 120 kV + Ag condition (Figure 3(a). In the lung, the absorbed dose also increased with effective energy from 80 to 135 kV, but decreased under the 120 kV + Ag condition despite its having the highest effective energy. In general, high‐energy X‐rays undergo less attenuation within the body, allowing more photons to penetrate more deeply into tissues, which may result in an increased absorbed dose. This trend is consistent with those in previous studies describing the relationship between X‐ray energy and dose distribution.

However, even under identical CTDIvol conditions, variations in the absorbed doses were observed. This finding suggests that the dose metrics displayed by CT systems alone may not be sufficient for accurate evaluation of organ doses.

### 4.2 Spectral changes and dose reduction with the silver filter

Under the 120 kV + Ag condition, the lung absorbed dose decreased despite having the highest effective energy, whereas the breast absorbed dose increased further under the same condition (Section 4.1). As shown in Figure 2, the addition of the Ag filter markedly reduces the low‐energy components and alters the spectral shape.

Low‐energy photons are more likely to be absorbed near the body surface and contribute substantially to dose.^11^ Therefore, the removal of these components likely led to the observed reduction in the lung absorbed dose. This finding is consistent with previous reports demonstrating dose reduction using spectral shaping filters such as tin filters. The breast absorbed dose, in contrast, continued to increase under the same condition; this tissue‐specific difference is considered further in Section 4.4 in relation to the energy dependence of the f‐factor.^7^

#### 4.2.1 Material‐dependent factors underlying the CTDIvol‐organ dose discrepancy

The discrepancy between the CTDIvol displayed by the CT system (measured in a PMMA phantom^17^) and the actual organ‐absorbed dose can be partly explained by differences in physical density and effective atomic number (Zeff) between PMMA and the tissues of interest. PMMA has a density of approximately 1.19 g/cm^3^ and a Zeff of approximately 6.6, values that are reasonably similar to those of breast tissue (density approximately 0.96‐1.02 g/cm^3^; Zeff approximately 7), but markedly different from those of lung tissue, whose density (approximately 0.26 g/cm^3^, owing to its air content) is roughly one‐fifth that of PMMA. Because Compton scattering, the dominant interaction at diagnostic CT energies, scales primarily with electron density (and hence physical density), the much lower density of lung tissue is expected to reduce the absolute energy deposited per unit mass compared with a PMMA phantom of the same CTDIvol, independent of beam quality. In contrast, because breast tissue density is closer to that of PMMA, the CTDIvol displayed by the system may be expected to track the breast‐absorbed dose more closely than the lung‐absorbed dose. This is broadly consistent with the present finding that the breast air kerma and absorbed dose were lower in absolute terms than those in the lung, while showing comparable relative trends with tube voltage.

The contribution of photoelectric absorption, which depends more strongly on Zeff and on photon energy, is expected to be small but non‐negligible at the effective energies used in this study (47‐89 keV), and may differ slightly between breast tissue and PMMA because of their difference in Zeff. This may contribute to part of the discrepancy between the effective‐energy‐ and mean‐photon‐energy‐based f‐factors observed in the breast (Section 4.3), although the present data do not allow this contribution to be separated from other sources of uncertainty. A full quantitative decomposition of these effects would require Monte Carlo simulation or direct measurement of CTDIvol in lung‐ and breast‐equivalent phantoms, which was beyond the scope of the present study.

### 4.3 Differences between effective energy and mean photon energy

In addition to the effective energy, we evaluated the mean photon energy. The relationship between the mean photon energy and absorbed dose differed from that observed using effective energy, and this difference was tissue‐dependent. In the lung, the absorbed dose decreased under the 120 kV + Ag condition regardless of whether effective energy or mean photon energy was used. In the breast, however, the two metrics led to opposite conclusions under the same condition: the absorbed dose increased when calculated using the effective energy but decreased when calculated using the mean photon energy (Figure 3(b).

Both the effective energy and the mean photon energy are single‐value representations of a polychromatic X‐ray spectrum, and neither fully captures the spectral distribution when used to derive the f‐factor. Effective energy is obtained from the aluminum HVL under a monoenergetic approximation, whereas the mean photon energy is derived by integrating the entire measured spectrum and may therefore reflect the spectral shape more directly; however, this does not by itself guarantee a more accurate absorbed‐dose estimate, because the f‐factor itself still treats the beam as monoenergetic regardless of which energy value is used.^13^

These results indicate that the discrepancy between the two metrics is not uniform across tissues, but instead emerges specifically in the breast under the Ag filter condition. This finding suggests that the choice of energy metric used to derive the f‐factor can meaningfully affect the estimated absorbed dose in certain tissues, and that this tissue‐specific sensitivity warrants further investigation, as discussed in Section 4.4.

### 4.4 Influence of f‐factor energy dependence on absorbed dose

As shown in Figure 4 and Table 3, differences were observed in the absorbed dose depending on whether the effective or mean photon energy was used, and the magnitude of this difference was tissue‐dependent. This is likely attributable to the energy dependence of the f‐factor.^8^, ^9^

**FIGURE 5:**
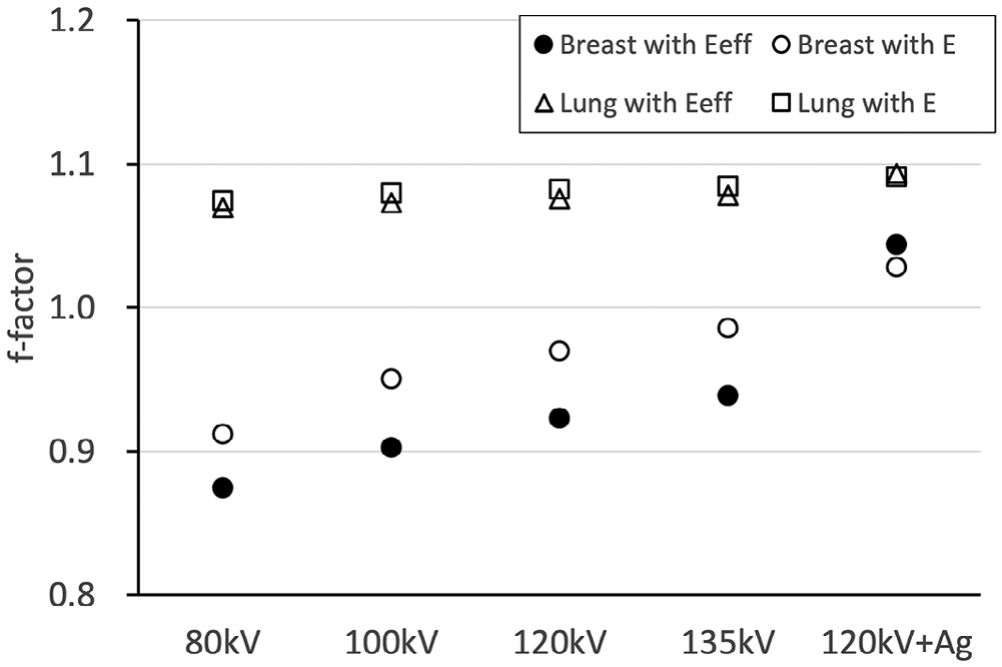
Relationship between photon energy and f‐factor for breast and lung tissues, illustrating the energy dependence of the f‐factor and its influence on absorbed dose estimation.

In the breast, the f‐factor increased from 0.87 (80 kV) to 0.94 (135 kV) and to 1.04 (120 kV + Ag) when calculated using the effective energy, and from 0.91 to 0.99 and to 1.03 when calculated using the mean photon energy. Because the breast air kerma decreased under the 120 kV + Ag condition (7.98 mGy, compared with 8.76 mGy at 135 kV), the small difference between the two f‐factors at this condition (1.04 versus 1.03) was sufficient to determine whether the resulting absorbed dose increased or decreased relative to the 135 kV condition. In the lung, by contrast, the f‐factor remained nearly constant across all conditions for both energy metrics (1.07‐1.09), which is consistent with the lung absorbed dose showing the same decreasing trend regardless of which energy metric was used. Under the Ag filter condition, the increase in photon energy altered the ratio of the mass energy‐absorption coefficients between the tissue and air, potentially resulting in f‐factor values exceeding unity and influencing the breast‐absorbed dose (Figure 5).

These findings highlight that the f‐factor's sensitivity to the choice of energy metric is not uniform across tissues, and that this sensitivity becomes practically relevant when the f‐factor approaches or exceeds unity, as observed in the breast under the Ag filter condition.

### 4.5 Organ‐specific differences (breast vs. lung)

Under all the conditions, the absorbed doses in the lungs were higher than those in the breasts. This may be attributed to the rotational irradiation geometry of CT and the contribution of scattered radiation within the body. Furthermore, the trend of the absorbed dose differed between the two tissues with increasing photon energy: in the lung, the absorbed dose closely tracked the air kerma and decreased under the 120 kV + Ag condition regardless of the energy metric used, whereas in the breast, the absorbed dose continued to increase under the same condition when using the effective energy, diverging from the air kerma trend.

Because the lungs are located deeper within the thorax, they may receive a greater contribution from scattered photons in addition to primary radiation. Consequently, the absorbed dose in the lungs may exceed that in superficial tissues, such as the breasts. This tendency is consistent with previous reports on organ dose distribution in chest CT.^10^ A related consideration is that, within a standard CTDI phantom, the dose at the peripheral positions is generally higher than that at the center for phantom diameters typical of the body region, although this relationship depends on the phantom size and the X‐ray energy. In the present anthropomorphic phantom, the breast is located closer to the body surface than the lung. This is broadly consistent with the peripheral‐to‐center dose gradient observed in CTDI phantom studies. However, the breast and lung also differ in depth, surrounding tissue composition, and density; therefore, the present results cannot be directly equated with a CTDI phantom dose profile. To provide context for this comparison, we additionally measured CTDI100 at the central and peripheral positions of a standard 32‐cm‐diameter PMMA body phantom under the same tube voltage conditions (without the Ag filter, which is only applicable in helical mode). The peripheral CTDI100 was approximately 1.9‐2.1 times higher than the central CTDI100 across the tested tube voltages, confirming the conventional peripheral‐to‐center dose gradient for this phantom size. The resulting weighted CTDI (CTDIw) was 4.8% to 7.2% higher than the CTDIvol value displayed by the CT system, indicating that the displayed CTDIvol reasonably approximated the measured value, with a small systematic underestimation.

### 4.6 Clinical implications

The present study demonstrates that organ‐absorbed doses vary depending on the tube voltage and beam‐shaping filters, even under identical CTDIvol conditions.

In particular, spectral shaping techniques such as Ag filters may contribute to dose reduction while simultaneously altering organ‐specific dose distributions. Therefore, the evaluation of radiation dose in CT should consider not only system‐displayed metrics but also beam quality and spectral characteristics.

## 5 LIMITATIONS

This study had some limitations. First, the measurements were performed using an anthropomorphic phantom and may not fully reflect patient‐specific variations in body habitus and tissue composition. In particular, the shape and position of the breasts vary considerably among individuals, which may affect dose distribution in clinical settings.

Second, this study was conducted using a single CT system, and it remains unclear whether similar results can be obtained using other CT systems or different beam‐shaping filters. The bow‐tie filter shape, the composition of the conventional and Ag beam‐shaping filters, and the automatic exposure control algorithm differ among CT manufacturers and models, and any of these factors could influence the measured spectra and the resulting f‐factor‐based dose estimates. Because the tissue‐dependent discrepancy between the effective‐energy‐ and mean‐photon‐energy‐based absorbed doses reported here was observed only under specific conditions (the breast under the 120 kV + Ag condition), confirming whether this discrepancy generalizes to other CT systems, tube voltage and filter combinations, and patient body sizes will require further multi‐scanner studies. Such validation was beyond the scope of the present single‐institution study and is left for future work.

## 6 CONCLUSION

This study investigated the effects of tube voltage and beam‐shaping filters on absorbed doses in the breasts and lungs during chest CT under identical CTDIvol conditions.

The absorbed dose generally increased with increasing effective energy in both tissues; however, with a silver filter, the removal of low‐energy components resulted in a reduction in the lung absorbed dose, whereas the breast absorbed dose continued to increase. Furthermore, when the mean photon energy was used instead of the effective energy, the absorbed dose decreased under the silver‐filter condition in both tissues, showing a different trend from that observed with the effective energy, particularly in the breast.

These findings indicate that the organ‐absorbed dose varies depending on beam quality, even under identical CTDIvol conditions, and that this variation is tissue‐dependent. Specifically, mean‐photon‐energy‐based absorbed doses in the breast were approximately 4%‐5% higher than effective‐energy‐based estimates under the standard (non‐filtered) conditions, whereas this relationship reversed under the 120 kV + Ag condition, with mean‐photon‐energy‐based doses approximately 1.6% lower than effective‐energy‐based estimates. Although the magnitude of this reversal was relatively small, it was consistent and reproducible across repeated measurements. Because the effective energy and the mean photon energy can lead to different, and in the breast even opposite, conclusions regarding the direction of dose change, the choice of energy metric used to convert air kerma to absorbed dose should be considered carefully, particularly when the f‐factor approaches or exceeds unity.

## AUTHOR CONTRIBUTIONS

**Yusuke Ito**: Conceptualization; methodology; investigation; data curation; formal analysis; writing—original draft. **Yusei Nishihara**: Methodology; investigation; writing—review and editing. **Masanao Kobayashi**: Methodology; investigation; writing—review and editing. **Tomoya Hibino**: Supervision. **Yasuki Asada**: Conceptualization; writing—review and editing; supervision.

## CONFLICT OF INTEREST STATEMENT

The authors declare no conflicts of interest.

## ETHICS STATEMENT

This study was conducted using an anthropomorphic phantom and did not involve human participants. Therefore, institutional review board approval was not required.

## Data Availability

The data that support the findings of this study are available from the corresponding author upon reasonable request.
